# 

**Published:** 1861-04

**Authors:** 


☞ MAD RIVER VALLEY SOCIETY.—The next quarterly meeting of "The Mad River Valley Dental Association" will be held at the Phillips House, in the city of Dayton, on the 2d April (Tuesday), at 2 P. M.

*Subjects for Discussion:*

1. "Periostitis."
2. "On the best mode of keeping natural and artificial teeth clean."
3. "On the best mode of separating teeth preparatory to plugging them."

G. L. PAINE, *Sec'y.*

☞ The second annual meeting of the Kentucky State Dental Association will convene in the City of Louisville, in the parlor of Masonic Temple, on Tuesday, April 9, 1861, at half-past 10 o'clock, A. M.

It is hoped that all interested will attend.

Respectfully, J. W. GRANT,
E. J. PECKOVER,
W. MUIR ROGERS,
*Executive Committee.*

### DENTAL REGISTER,

### OF THE WEST.

**Issued Monthly, at $3 a year in advance.**

DEVOTED TO THE INTERESTS OF THE DENTAL PROFESSION.

**Edited by J. TAFT and GEO. WATT.**

The Volume commences in January and closes in December.

The Register being issued monthly is always fresh, therefore indispensable to the practitioner who desires to keep posted up with the new inventions and improvements which are daily developed. Neither expense nor effort will be spared to give value and variety to its contents. Articles will be illustrated with well executed engravings, whenever they will add to the interest or assist in explanation.

Its extensive circulation and frequency of issue makes it the BEST DENTAL ADVERTISING MEDIUM in the United States.

☞ CONTRIBUTIONS SOLICITED.

Address Original Communications to **Geo. Watt, Xenia, O.**

Exchanges to **J. Taft**, or **Dental Register, Cincinnati, O.**

Business Gommunications, to

**JNO. T. TOLAND, Publisher and Proprietor,**
38 West Fourth Street, Cincinnati, O.

Communications must reach the Editor as early as the 15th, to insure insertion in the next issue.

